# Supplementary material for: Lockdowns exert selection pressure on overdispersion of SARS-CoV-2 variants
Source: Epidemics. 2022 Sep;40:100613. doi: 10.1016/j.epidem.2022.100613 (PMC9338171; doi:10.1016/j.epidem.2022.100613)
Supplement: MMC S1 — Supplementary analyses. [file mmc1.pdf]

Supporting Information: Lockdowns exert selection pressure on overdispersion of SARS-CoV-2 variants

Bjarke Frost Nielsen,[1,*] Andreas Eilersen,[1,†] Lone Simonsen,[2,‡] and Kim Sneppen[1,§]
[1] *Niels Bohr Institute, University of Copenhagen, Blegdamsvej 17, 2100 Copenhagen, Denmark.*
[2] *Department of Science and Environment, Roskilde University, Universitetsvej 1, 4000 Roskilde, Denmark.*
(Dated: June 23, 2022)

S1. INTERVENTIONS TARGETING TRANSMISSION RISK

In the main text we have primarily considered the evolutionary pressures exerted by mitigation strategies which rely on reductions in social connectivity, such as lockdowns. However, other mitigation strategies may rely not on limiting the number of distinct individuals that a person interacts with, but rather on decreasing the transmission rate when contacts *do* occur. Examples of such strategies include the use of face masks, which work primarily by decreasing the number of emitted virions, and physical distancing which exploits the decrease in viral concentration as distance to the source is increased. In Fig. S1, we consider two scenarios in which a more homogeneous variant with dispersion factor $k = 0.2$ emerges in the background of the $k = 0.1$ ancestral strain. Note that the curves in Fig. S1 show the relative *share* of infections owing to the emerging variant and not the absolute incidence. In both of the cases studied, social connectivity is initially quite restrictive, with each person allowed only 10 contacts. At time $t = 25$days, restrictions are relaxed, simulated by increasing average connectivity to 50. This is where the two scenarios diverge: in scenario 1 (red curve), the infection risk *per encounter* is halved, while in scenario 2 (yellow) it stays the same. It is of course expected that halving the infection risk will cause a smaller overall epidemic, but what is not obvious is how it will affect the competition between two variants differing only in their level of overdispersion. As one would expect from Fig. 3 of the main text, the competitive advantage of the new variant is reduced by going from 10 to 50 social connections, as is reflected by both of the curves in Fig. S1 bending off at $t = 25$days. However, more notable is the fact that the competitive advantage of the new variant is substantially reduced when restrictions are put in place which decrease the infection rate. The conclusion is thus that heterogeneous variants are particularly vulnerable to lockdown-type interventions, where social network size is reduced, while more homogeneous variants are more susceptible to interventions which reduce infectiousness during each encounter.

* bjarkenielsen@nbi.ku.dk
† andreaseilersen@nbi.ku.dk
‡ lonesimo@ruc.dk
§ sneppen@nbi.dk

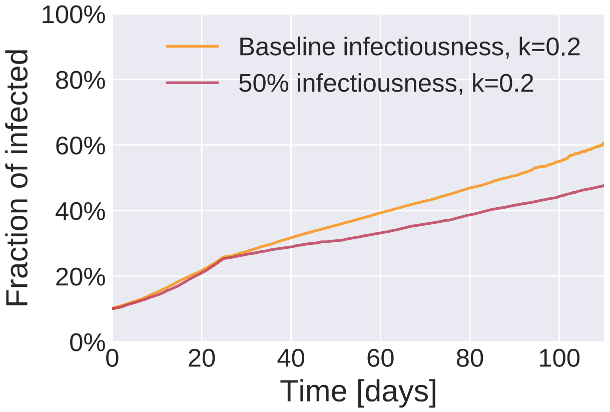

FIG. S1. **Re-opening society while putting other restrictions in place.** The plot shows the relative abundance of a new, more homogeneous variant ($k = 0.2$) as a function of time. At time $t = 0$, the new variant makes up 10% of infections, while the rest are due to the more heterogeneous "old" variant ($k = 0.1$). Until time $t = 25$ a partial lockdown is in place, modeled by restricting personal contact networks to only 10 persons on average. At $t = 25$days, society is partially opened, simulated by allowing contact with 50 different persons. The red line represents a scenario where the opening of society is accompanied by other restrictions, reducing the infection risk *per encounter* by half. As such, the diversity of contacts encountered is increased, but the infection risk per encounter is decreased. In the scenario shown by the yellow line, infection risk per encounter is unaltered. Clearly, these interventions negatively affect the competitive advantage of the more homogeneous variant.

In Fig. S2 we show the absolute incidence of the two variants in another simulation experiment where the two types of interventions are in force simultaneously. Initially, only the (partial) lockdown is in force, with social connectivity restricted to 10 persons. At $t = 35$ days, another non-pharmaceutical intervention which reduces the transmission risk per encounter by 20% is put in force. This is seen to reduce the more homogeneous emerging variant to marginal spread while the ancestral strain starts to decline.

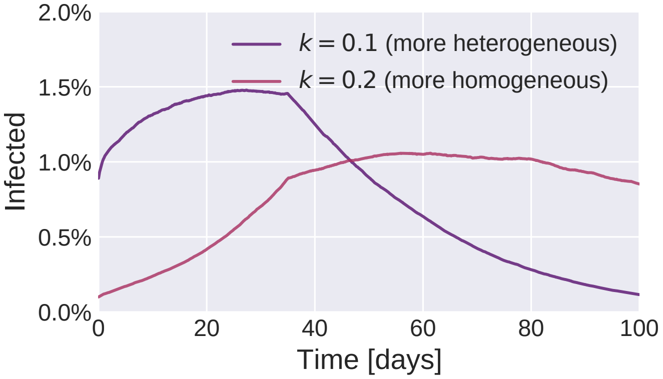

FIG. S2. Two variants with dispersion parameters $k = 0.1$ and $k = 0.2$ spread in a socially restricted society with a personal contact network size of 10. From time $t = 35$days, non-pharmaceutical interventions are introduced which reduce the rate of transmission by 20%. This is enough to reduce the more homogeneous ($k = 0.2$) variant to marginal growth, while the heterogeneous variant dies out.

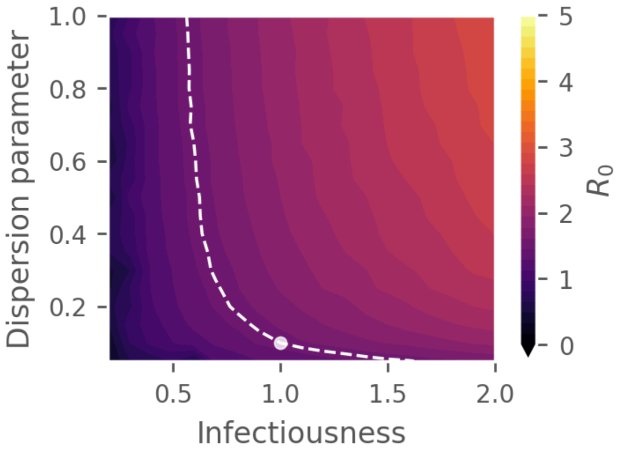

FIG. S3. **Fitness of variants in a scale free contact network.** Here we probe the R_0 attained by variants differing in their degree of biological transmissibility and overdispersion, when spreading on a contact network with degree distribution given by Eq. (S1). All connectivities have been scaled such that the mean connectivity is 10. The corresponding main text figure is Fig. 3B.

S2. PARTIAL LOCKDOWNS WITH RESIDUAL HIGH-CONNECTIVITY INDIVIDUALS

Even in a (partial) lockdown scenario, it is expected that some individuals (such as essential workers) may maintain a relatively high connectivity. To model such a scenario, we assume an approximately scale free network with a degree distribution of the form

$$p(c) \propto \frac{c}{(c+a)^{3.5}}, \tag{S1}$$

which is asymptotically scale free with a power law exponent of -2.5, leading to a wide distribution of connectivities.

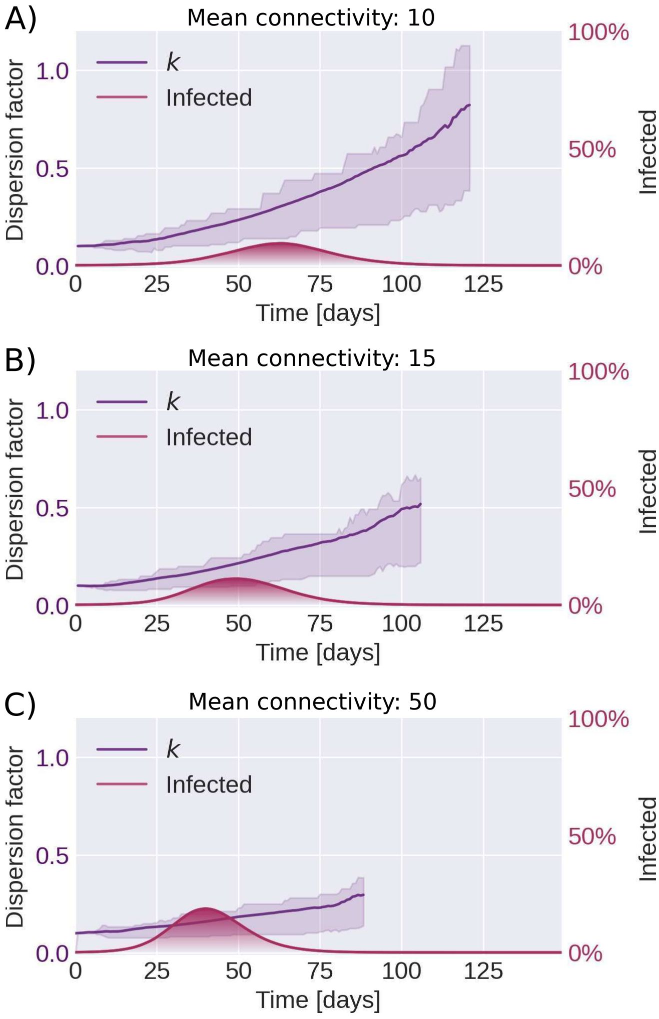

FIG. S4. **Overdispersion evolving in a scale-free network.** Employing the same evolutionary algorithm as in Fig. 4 of the main text, a similar – albeit somewhat attenuated – tendency towards decreasing overdispersion (i.e. higher value of k) is seen. The network employed here has a degree distribution given by Eq. (S1), with all degrees scaled so as to give the desired mean connectivity (10 in panel A, 15 in panel B and 50 in panel C). Each panel represents an average over 5 simulations.

In Fig. S4, we let the dispersion parameter k evolve as in Figure 4 of the main text, but in a network following the above degree distribution. We perform the simulation for three different values of mean connectivity, to probe the sensitivity to contact reduction. The mean connectivity is modulated by rescaling all degrees drawn from the distribution (S1) – as opposed to by a cut-off – ensuring that high-connectivity nodes continue to exist.

We observe that the tendency towards developing a higher k value (i.e. towards more homogeneous spread-

ing) is somewhat attenuated by the presence of high-connectivity nodes, when compared to the main text figure 4. However, the overall pattern persists, revealing that it is robust even in networks with wide degree distributions.

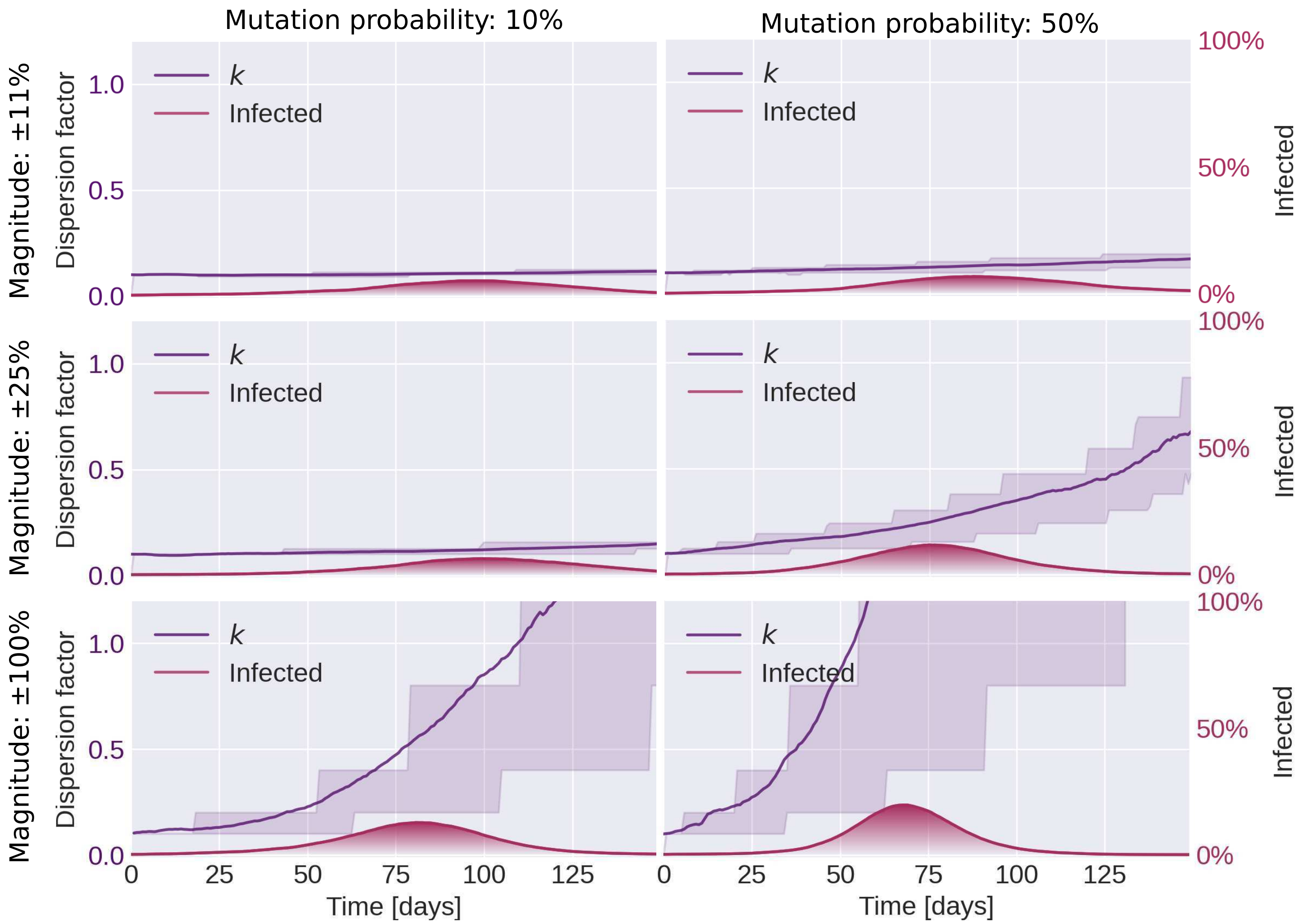

FIG. S5. **Overdispersion evolving in a scale-free network.** Employing the same evolutionary algorithm as in Fig. 4 of the main text, but varying the mutation rate (parameterised as a probability of a mutation occurring within each host) and the magnitude of mutations affecting the dispersion parameter k. When the magnitude is e.g. 25%, a mutation affecting k will have equal probability of increasing or decreasing it by 25% in that host.
